# Purinergic receptor-induced Ca^2+^ signaling in the neuroepithelium of the vomeronasal organ of larval *Xenopus laevis*

**DOI:** 10.1007/s11302-013-9402-3

**Published:** 2013-11-23

**Authors:** Katarina Dittrich, Alfredo Sansone, Thomas Hassenklöver, Ivan Manzini

**Affiliations:** 1 Institute of Neurophysiology and Cellular Biophysics, University of Göttingen, Humboldtallee 23, 37073 Göttingen, Germany; 2 Center for Nanoscale Microscopy and Molecular Physiology of the Brain (CNMPB), 37073 Göttingen, Germany

**Keywords:** P2Y receptors, Olfactory system, Calcium imaging, Pharmacological characterization

## Abstract

Purinergic signaling has considerable impact on the functioning of the nervous system, including the special senses. Purinergic receptors are expressed in various cell types in the retina, cochlea, taste buds, and the olfactory epithelium. The activation of these receptors by nucleotides, particularly adenosine-5′-triphosphate (ATP) and its breakdown products, has been shown to tune sensory information coding to control the homeostasis and to regulate the cell turnover in these organs. While the purinergic system of the retina, cochlea, and taste buds has been investigated in numerous studies, the available information about purinergic signaling in the olfactory system is rather limited. Using functional calcium imaging, we identified and characterized the purinergic receptors expressed in the vomeronasal organ of larval *Xenopus laevis*. ATP-evoked activity in supporting and basal cells was not dependent on extracellular Ca^2+^. Depletion of intracellular Ca^2+^ stores disrupted the responses in both cell types. In addition to ATP, supporting cells responded also to uridine-5′-triphosphate (UTP) and adenosine-5′-O-(3-thiotriphosphate) (ATPγS). The response profile of basal cells was considerably broader. In addition to ATP, they were activated by ADP, 2-MeSATP, 2-MeSADP, ATPγS, UTP, and UDP. Together, our findings suggest that supporting cells express P2Y_2_/P2Y_4_-like purinergic receptors and that basal cells express multiple P2Y receptors. In contrast, vomeronasal receptor neurons were not sensitive to nucleotides, suggesting that they do not express purinergic receptors. Our data provide the basis for further investigations of the physiological role of purinergic signaling in the vomeronasal organ and the olfactory system in general.

## Introduction

The vomeronasal system is present as a discrete sensory system only in tetrapods. It first appeared in amphibians ([1, 2] but see [3]), and it is present from larval to adult stages in most amphibian species [4]. The vomeronasal organ (VNO) is the peripheral sensory organ of the vertebrate accessory olfactory system [5]. The VNO has been shown to mediate the detection of pheromones [4, 5], but in *Xenopus* its function still remains to be elucidated. It consists of a neuroepithelium containing the same three main cell types as the vertebrate main olfactory epithelium (MOE): (1) receptor neurons which transmit the sensory information from the periphery to the accessory olfactory bulb in the brain, (2) supporting cells (SCs) which share common properties with glial and epithelial cells, and (3) basal cells (BCs), stem cells which maintain the regenerative capacity of the organ. Specifically, the VNO of larval *Xenopus laevis* is made of microvillous vomeronasal receptor neurons (VRNs), ciliated SCs, and a population of BCs. The *Xenopus* MOE, in contrast, consists of microvillous and ciliated olfactory receptor neurons (ORNs), ciliated and secretory SCs, and a population of BCs; for a more detailed overview of the cellular organization of the *Xenopus* olfactory organ, see [1, 6].

It has been shown that SCs and BCs in the *Xenopus* and mouse MOE express purinergic receptors [7–10]. In both species, application of nucleotides to the MOE induces strong wave-like intracellular Ca^2+^ ([Ca^2+^]_i_) increases in SCs that propagate from the apical to the basal part of the MOE [8, 11], suggesting that they are a common feature of the vertebrate MOE. In *Xenopus*, these Ca^2+^ waves have been shown to be triggered by P2Y_2_/P2Y_4_-like purinergic receptors [8]. Based on these results, it has been hypothesized that SCs could constitute a transepithelial information path that carries information from the apical to the basal part of the MOE. Nucleotides released from damaged or dying ORNs could initiate Ca^2+^ waves in nearby SCs, which then, carry this information to the BCs. The elevated [Ca^2+^]_i_ in the basal endings of SCs could then stimulate nucleotide release in the basal cell layer, thereby activating proliferation of new neurons (for a review see [12]). In fact, it has been shown that BCs in *Xenopus* express multiple P2Y receptors and that purinergic signaling influences the cell turnover in the MOE [9]. Also, in the mouse MOE activation of purinergic receptors induces proliferation and neuronal differentiation [13, 14]. It is known that purinergic signaling is generally involved in embryonic and adult neurogenesis [15, 16], and also cells of neurogenic brain areas in *Xenopus* have been shown to express purinergic receptors [17]. In contrast to SCs and BCs, big differences regarding the expression of purinergic receptors exist between murine and amphibian ORNs. While murine ORNs have been shown to express P2X and P2Y receptors [10, 18], ORNs of *Xenopus* do not express purinergic receptors [7, 8]. The activation of purinergic receptors in mouse ORNs has been shown to reduce their responsiveness to odorants [10].

In contrast to the MOE, very little is known about purinergic signaling in the VNO. To our knowledge, there are only two studies describing purinergic receptors in the VNO. These studies showed that murine VRNs express P2X receptors [18, 19], and SCs in the rat VNO express P2Y receptors [18]. Activation of P2X receptors in the mouse VNO leads to an increase of the chemosensory response in VRNs [19].

The present study was aimed to functionally identify the purinergic receptor subtypes expressed in the various cell types of the epithelium of the VNO of larval *X. laevis*. We provide evidence that SCs express P2Y_2_/P2Y_4_-like purinergic receptors and BCs express multiple P2Y receptors. Vomeronasal receptor neurons are not sensitive to nucleotides, suggesting that they do not express purinergic receptors. Thus, the purinergic system of the *Xenopus* vomeronasal epithelium appears to be very similar to the purinergic system of the MOE [8, 9], suggesting that purinergic receptors might have similar functional effects in both olfactory subsystems.

## Material and methods

### Preparation of acute Fluo-4 stained slices of the vomeronasal epithelium

Larval *X. laevis* (stages 51 to 54; staged after [20]) were cooled in iced water to produce complete immobility and killed by transection of the brain at its transition to the spinal cord, as approved by the Göttingen University Committee for Ethics in Animal Experimentation. A block of tissue containing the olfactory system was cut out and cut horizontally into 130–140 μm thick slices with a vibrotome (VT 1200S, Leica, Bensheim, Germany). Tissue slices were loaded with the Ca^2+^-sensitive dye Fluo-4/AM (Molecular Probes, Leiden, The Netherlands) as described in previous work (see [8]). Calcium imaging of nucleotides responses was carried out as described below.

### Ca^2+^ imaging and data evaluation

Changes of intracellular calcium concentrations of cells of the epithelium of the VNO were monitored using an upright confocal laser-scanning microscope (LSM 780/Axio Examiner, Zeiss, Jena, Germany). Fluorescence images (excitation at 488 nm; emission >495 nm) of the VNO were acquired at 1 Hz, with about 5–10 images taken as control before the onset of stimulus delivery. The thickness of the optical slices excluded fluorescence detection from more than one cell layer. Image analysis was performed using custom programs written in MATLAB (MathWorks, Natick, USA). To facilitate the selection of regions of interest, a “pixel correlation map” was obtained (see [21]). The fluorescence changes for individual cells are given as Δ*F*/*F* values. For more detailed information, see our previous work [8]. Averaged data are presented as mean ± standard error of the mean (SEM). Statistical significance was determined by paired Student’s *t* test. Maximum amplitude values of purinergic agonist applications were normalized to an ATP control application.

### Solutions and stimulus application

Standard bath solution consisted of (in millimolar): 98 NaCl, 2 KCl, 1 CaCl_2_, 2 MgCl_2_, 5 glucose, 5 Na-pyruvate, 10 hydroxyethyl piperazineethanesulfonic (HEPES), 230 mOsmol/l, pH 7.8. Ca^2+^-free bath solution consisted of (in millimolar): 98 NaCl, 2 KCl, 2 MgCl_2_, 5 glucose, 5 Na-pyruvate, 10 HEPES, 2 EGTA, 230 mOsmol, pH 7.8. High K^+^ bath solution consisted of (in millimolar): 17 NaCl, 80 KCl, 1 CaCl_2_, 2 MgCl_2_, 5 glucose, 5 Na-pyruvate, 10 HEPES, 230 mOsmol/l, and pH 7.8. The BrdU; uridine-5′-diphosphate (UDP); UTP; adenosine; adenosine-5′-diphosphate (ADP); ATP; 2-methylthio-ATP (2-MeSATP); ATPγS; 2-methylthio-ADP (2-MeSADP); suramin, 3′-O-(4-benzoyl)benzoyl ATP (BzATP), α,β-methylene ATP (α,β-meATP); β,γ-methylene ATP (β,γ-meATP), cyclopiazonic acid (CPA), and bath solution chemicals were purchased from Sigma (Seelze, Germany). Bath solution was applied by gravity feed from a storage syringe through a funnel drug applicator to the recording chamber. The purinergic agonists were pipetted directly into the funnel without stopping the flow. During each application, 1 ml of agonist solution was applied into the funnel within about 2 s. Outflow was through a syringe needle placed close to the basal regions of the vomeronasal epithelium. Suramin and CPA were added to the bath solution where indicated. The minimum interstimulus interval between agonist applications was at least 3 min in all of the experiments. The minimum wash time after application of Ca^2+^-free bath solution, CPA and suramin was 3–6 min.

### Preparation of backfilled slices, BrdU injections, and immunohistochemistry

To backfill VRNs, larval *X. laevis* (stages 51 to 54) were anesthetized in 0.02 % MS-222 (Ethyl 3-aminobenzoate methanesulfonate; Sigma), and their olfactory nerves were transected. Biocytin (ε-biotinoyl-l-lysine, Molecular Probes) or micro-Ruby (Life Technologies, Darmstadt, Germany) crystals were put into the lesioned nerve, and the lesion was closed with tissue adhesive (Histoacryl L, Braun, Tuttingen, Germany). After 1 h the animals were killed, and a tissue block (see above) was cut out. Tissue blocks of micro-Ruby backfilled animals were then sliced and stained with Fluo-4 as described above. To visualize the micro-Ruby-stained VRNs, we acquired fluorescence images of the VNO using the same confocal laser-scanning microscope as for the calcium imaging experiments (excitation at 561 nm; emission >565 nm). The combination of VRN-staining with micro-Ruby on the one hand and calcium imaging of nucleotide responses on the other allowed to differentiate between responses of neuronal (VRNs) and non-neuronal (BCs and SCs) cells. To test the viability of the VRNs, we applied high K^+^ bath solution. All cells responding with a transient increase of fluorescence were considered healthy.

To stain cells in the S phase of their cell cycle, we injected 200 μM 5-bromo-2′-deoxyuridine (BrdU, Sigma) intraperitoneally. After 2 h, the animals were sacrificed and a tissue block containing the vomeronasal organ and the olfactory bulb was removed.

For immunohistochemistry, the tissue blocks were fixed in 4 % formaldehyde, washed in phosphate-buffered saline (PBS), embedded in 5 % low melting point agarose (Sigma), and sectioned on the vibratome at 70 μm. Sections were washed in PBS containing 0.2 % Triton X-100 (PBST), and non-specific binding was blocked with 2 % normal goat serum (NGS; ICN, Aurora, OH, USA) in PBST for 1 h. Slices of BrdU-treated animals were incubated in 1 N HCl at 37 °C for 45 min to denature DNA. Slices were incubated overnight at 4 °C with primary antibodies [anti *X. laevis* cytokeratin II (1h5, monoclonal, derived from mouse), or anti BrdU (B2531, monoclonal, derived from mouse, Sigma), diluted in 2 % NGS/PBST, all 1:1,000]. The *X. laevis* cytokeratin type II antibody developed by Michael Klymkowsky was obtained from the Developmental Studies Hybridoma Bank developed under the auspices of the NICHD and maintained by the University of Iowa, Department of Biological Sciences, Iowa City, IA 52242. Primary antibodies were washed off with PBS, and Alexa 546 conjugated streptavidin (Molecular Probes) was applied at a final concentration of 5 μg/ml in PBST for 5 h to visualize the biocytin backfilled VRNs. Slices were repeatedly rinsed in PBS, and Alexa 488 conjugated goat anti mouse secondary antibody (Molecular Probes) was applied at a dilution of 1:250 in 1 % NGS/PBS for 1.5 h. The secondary antibodies were washed off in several changes of PBS. Cell nuclei were stained with propidium iodide (1 μg/ml). Slices were transferred to slides, mounted in mounting medium (Dako, Hamburg, Germany) and visualized using a laser-scanning confocal microscope (Zeiss LSM 510/Axiovert 100 M, Jena, Germany).

## Results

### Cellular organization of the epithelium of the vomeronasal organ

**Fig. 1:**
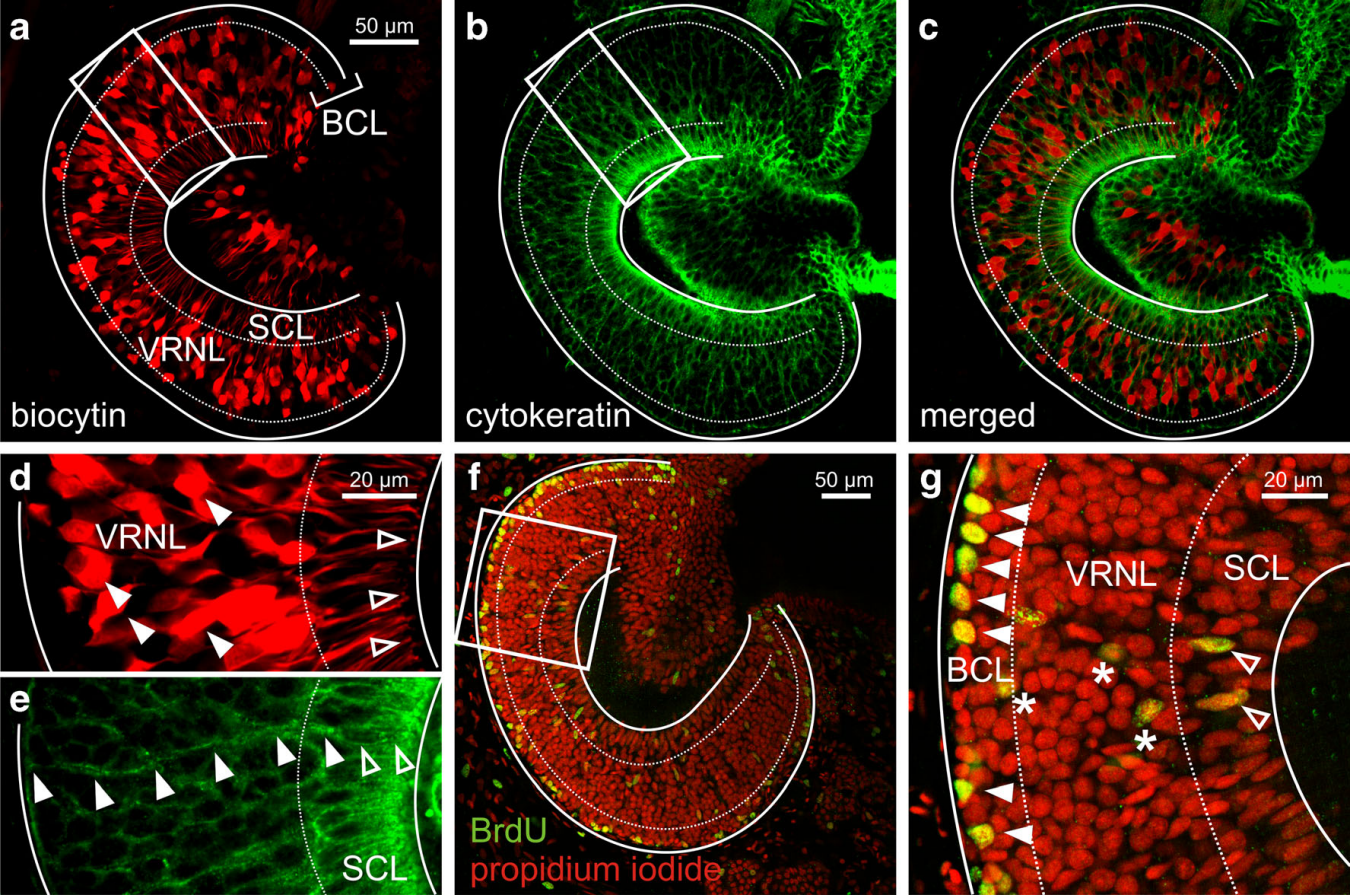
Morphological organization of the epithelium of the vomeronasal organ of larval *Xenopus laevis*. **a** Slice of a VNO with biocytin-streptavidin-stained VRNs (*SCL* supporting cell layer; *VRNL* vomeronasal receptor neuron layer; *BCL* basal cell layer). **b** Supporting cell staining with an antibody against cytokeratin type II of the same slice. **c** Overlay of VRNs (**a** red fluorescence) and SCs (**b** green fluorescence). Basal cells are biocytin-streptavidin and cytokeratin II negative and are located in the BCL. **d** and **e** Higher magnification of the region delineated by a white rectangle in **a** and **b**, respectively. The filled arrowheads in **d** indicate the somata of some VRNs. The *open arrowheads* indicate the apical endings of some VRNs (knobs). The *open arrowheads* in **e** indicate the soma of a SC, the *filled arrowheads* point at the basal process of the SC spanning the whole epithelium of the VNO. **f** Slice of another VNO with propidium iodide-stained cell nuclei (*red fluorescence*) and BrdU-positive cells (*green fluorescence*). The vast majority of BrdU positive cells are confined to the BCL of the VNO. **g** Higher magnification of the region delineated by a white rectangle in **f**. The *filled arrowheads* indicate BrdU-positive cells in the basalmost part of the epithelium. *Asterisks* indicate BrdU-positive cells in the VRNL, most probably maturing VRNs. *Open arrowheads* point at BrdU-positive SCs (see typical elongated nucleus)

To visualize the various cell types of the neuroepithelium of the VNO of larval *X. laevis*, we backfilled VRNs with biocytin through the olfactory nerve (Fig. 1a), and stained SCs with antibodies against cytokeratin (Fig. 1b). In the MOE of larval *Xenopus*, antibodies against cytokeratin exclusively label SCs [8]. When merging both pictures (Fig. 1c) the stratification of the OE becomes evident. The apical layer of the vomeronasal epithelium consists of somata of SCs (Fig. 1c and e) and dendrites of VRNs (Fig. 1c and d). The SC somata form the columnar supporting cell layer (SCL). Supporting cells extend thin cytoplasmatic appendages across the entire vomeronasal epithelium that terminate in endfoot-like structures at the level of the basal lamina (Fig. 1e). Somata of VRNs reside in the middle part of the epithelium and form the vomeronasal receptor neuron layer (VRNL). The most basal layer of the epithelium (basal cell layer, BCL) is devoid of mature VRNs and harbors vomeronasal stem cells, so-called BCs. To visualize proliferative BCs, we adopted a BrdU incorporation assay (Fig. 1f). A higher magnification of a part of the same epithelium as shown in Fig. 1f clearly depicts a number of BrdU-positive BCs (Fig. 1g). Most BrdU-positive cells are in the basalmost layer of the epithelium. The few BrdU-positive cells in the VRNL most probably are immature, migrating VRNs, and the two BrdU-positive cells in the SCL, on the other hand, could represent newly divided SCs (Fig. 1g).

### ATP-induced increases of [Ca^2+^]_i_ in cells of the vomeronasal epithelium

**Fig. 2:**
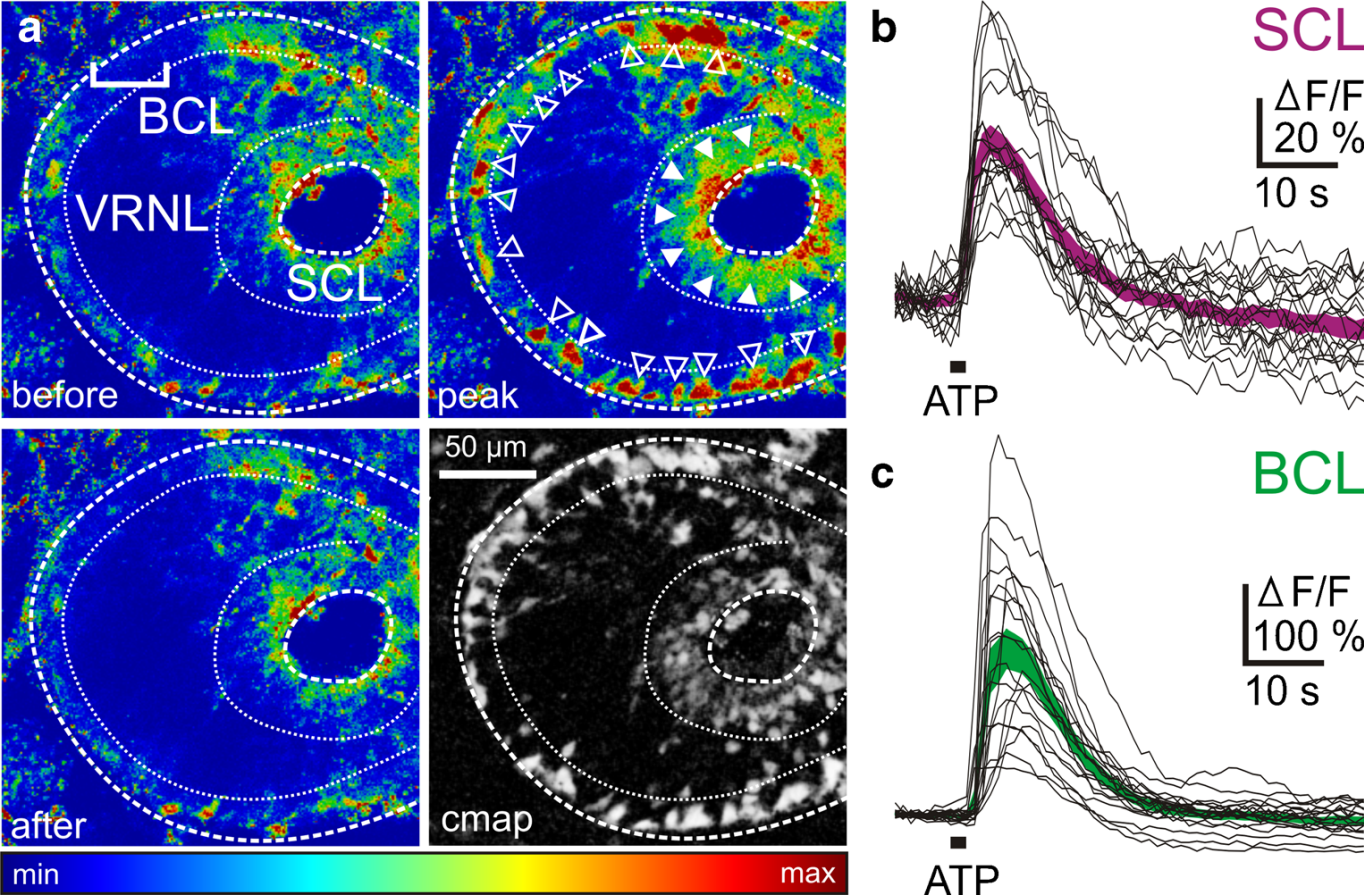
ATP-induced [Ca^2+^]_i_ increases in supporting and basal cells of the epithelium of the VNO of larval *Xenopus laevis*. **a** Pseudocolored image of an acute VNO slice stained with the Ca^2+^ indicator dye Fluo-4 (*SCL* supporting cell layer; *VRNL* vomeronasal receptor neuron layer; *BCL* basal cell layer). The *upper left*-*hand image* was acquired before application of ATP. Application of ATP-induced [Ca^2+^]_i_ transients in cells of the BCL (*open arrowheads*) and SCL (*filled arrowheads*). No apparent changes in Ca^2+^-dependent fluorescence in cells of the VRNL (*upper right*-*hand image*). The lower left-hand image was taken after return to the base line fluorescence. A pixel correlation map (see Materials and methods for details) of the same slice is depicted in the lower right-hand image. Responsive cells appear bright on dark background. **b** ATP-induced [Ca^2+^]_i_ transients of individual cells from the SCL (black traces). The *magenta*-*colored area* gives the mean [Ca^2+^]_i_ transients ± SEM of all individual SCs (*n* = 20). **c** ATP-induced [Ca^2+^]_i_ transients of individual cells from the BCL (*black traces*). The *green*-*colored area* gives the mean [Ca^2+^]_i_ transients ± SEM of all individual BCs (*n* = 20)

Application of ATP (100 μM) to acute slices of the VNO stained with Fluo-4 led to transient cellular Ca^2+^ responses, mainly in the SCL and the BCL. Thereby, the responses of SCs exhibited a wave-like pattern propagating from the apical to the basal part of the epithelium (data not shown). The pseudocolored images of Fig. 2a show a slice of the VNO before, at the peak of and after recovery of the ATP-induced response. The correlation map (see Material and methods) in the lower right corner shows all responsive cells of this slice. The response time courses of all ATP-sensitive cells of the SCL and the BCL of this slice are plotted in Fig. 2b and c, respectively. Virtually identical results were obtained with all VNO slices tested for their responsiveness to ATP. Cells in the VRNL showed virtually no response to ATP, indicating that neuronal cells of the VNO do not express purinergic receptors.

**Fig. 3:**
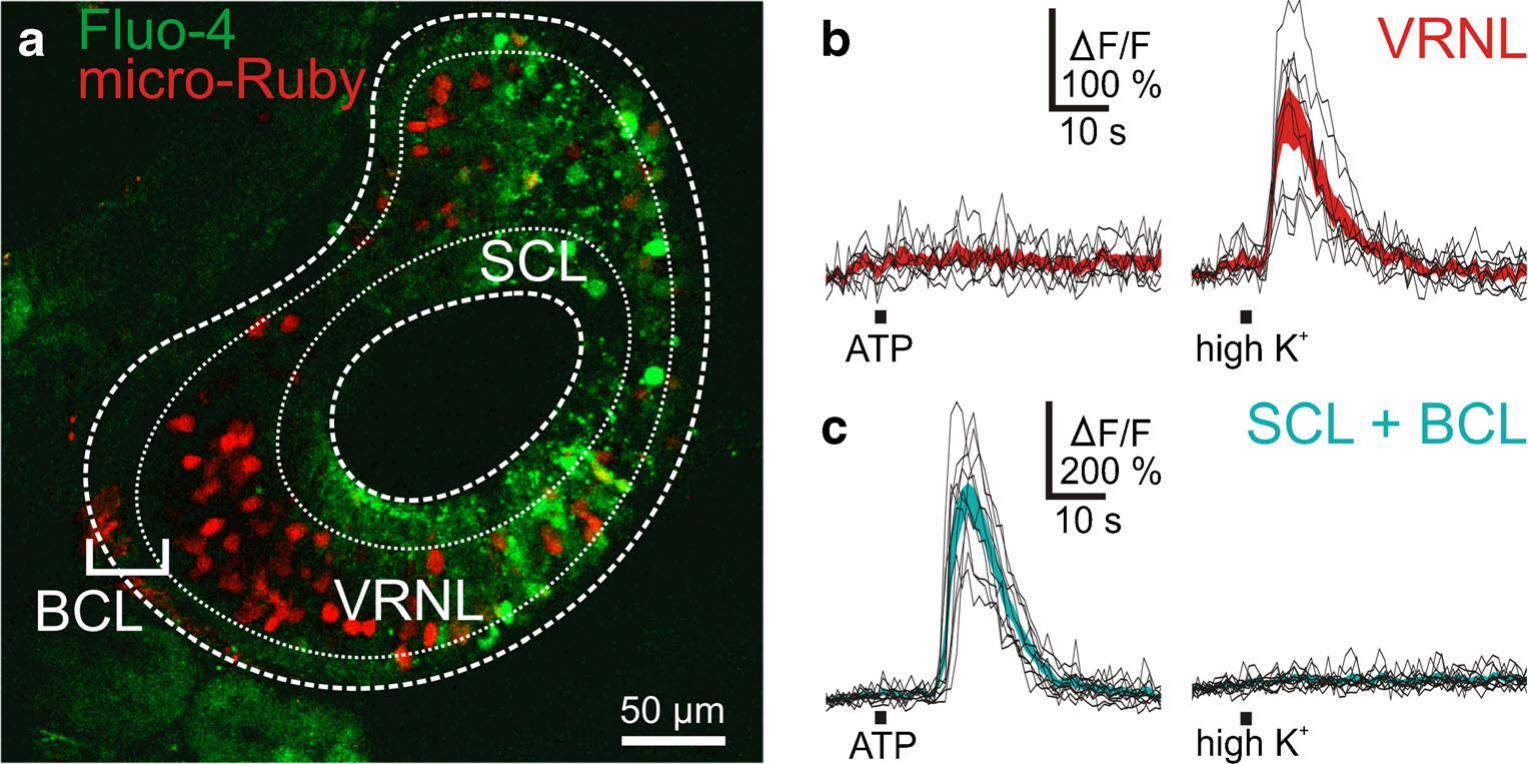
Vomeronasal receptor neurons do not respond upon application of ATP. **a** A micro-Ruby backfilled and Fluo-4 stained acute slice of the vomeronasal organ (*red* VRNs; *green* Fluo-4 stained cells; *SCL* supporting cell layer; *VRNL* vomeronasal receptor neuron layer; *BCL* basal cell layer). **b** ATP and high K^+^-induced [Ca^2+^]_i_ transients of individual micro-Ruby backfilled cells (i.e., VRNs; black traces). The *red*-*colored areas* give the mean [Ca^2+^]_i_ transients ± SEM of all individual VRNs (*n* = 9). The VRNs did not respond upon application of ATP, but all responded upon stimulation with high K^+^ solution. **c** ATP and high K^+^-induced [Ca^2+^]_i_ transients of individual non-neuronal cells (i.e., micro-Ruby negative cells from the SCL and BCL). The *cyan*-*colored* areas give the mean [Ca^2+^]_i_ transients ± SEM of all individual non-neuronal cells (*n* = 11). Non-neuronal cells responded upon application of ATP, but did not respond upon stimulation with high K^+^

To substantiate this indication, in a set of experiments, VRNs were backfilled with micro-Ruby previous to tissue slicing and Fluo-4 staining (see Materials and methods). Figure 3a shows an example of a micro-Ruby backfilled and Fluo-4 stained acute slice of the VNO. The red (micro-Ruby) VRNs can easily be distinguished from the other cells. Figure 3b and c, respectively, show responses to ATP and to application of high-K^+^ bath solution of neuronal (VRNs) and non-neuronal (SCs and BCs) cells. The two cell populations showed an opposite response pattern. Vomeronasal receptor neurons responded to the application of high K^+^ bath solution, but did not respond to ATP (100 μM), non-neuronal cells of the BCL and SCL responded to ATP but not to high K^+^ solution. Virtually identical results were obtained with all micro-Ruby backfilled slices (39 VRNs and 42 non-neuronal cells, 4 VNO slices). This clearly suggests that BCs and SCs, but not VRNs, express purinergic receptors.

### Pharmacological characterization of the purinergic receptor(s) expressed in the vomeronasal organ

**Fig. 4:**
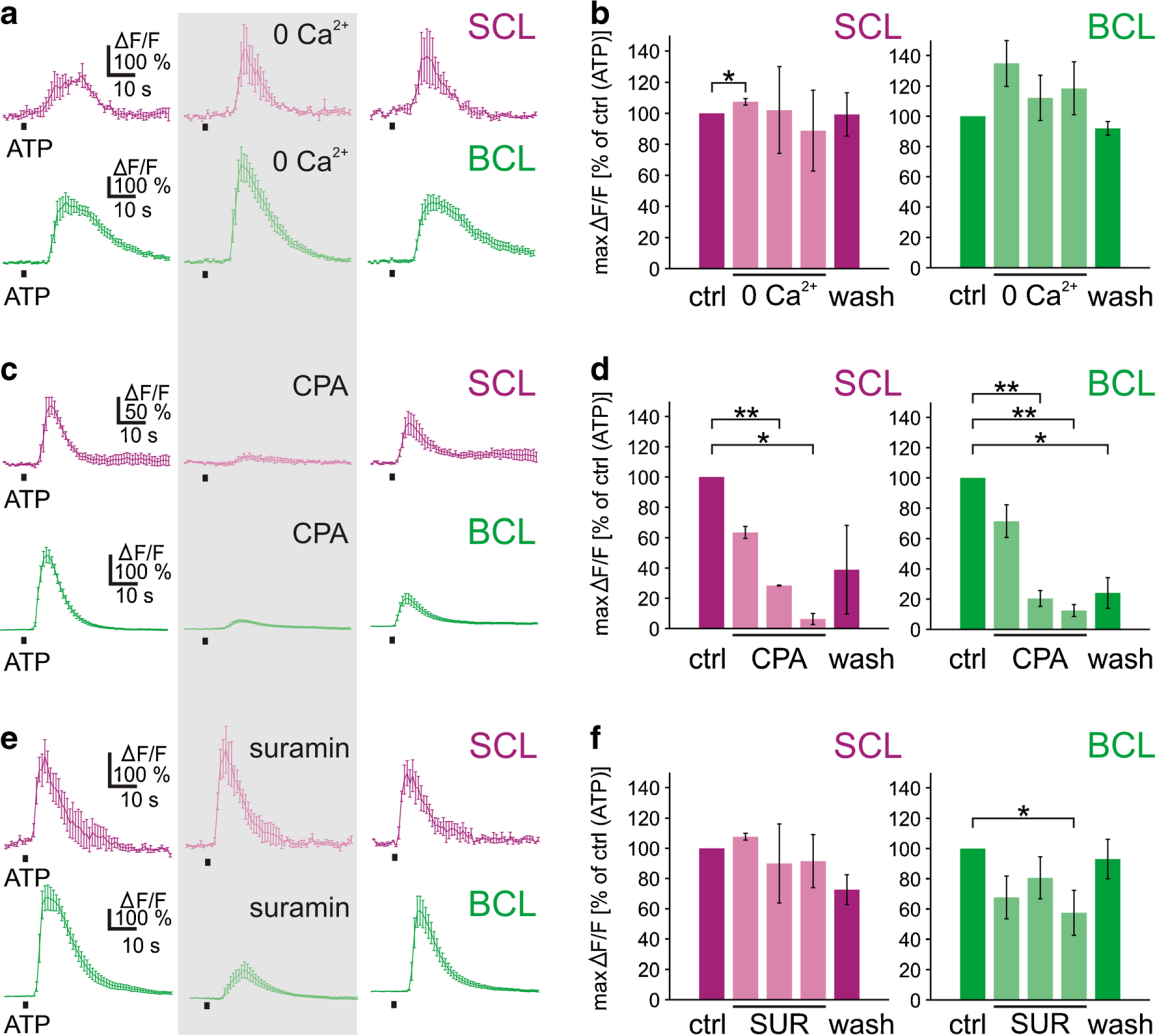
ATP-induced [Ca^2+^]_i_ increases of supporting and basal cells do not depend on extracellular Ca^2+^ but are dependent of store depletion, and are differentially affected by the purinergic antagonist suramin. **a** ATP-induced [Ca^2+^]_i_ increases of SCs (*magenta*) and BCs (*green*; mean response ± SEM of all ATP-responsive cells of an acute slice of the VNO; *SCL* supporting cell layer; *BCL* basal cell layer) persisted in Ca^2+^-free bath solution (*gray*-*shaded rectangle*; ATP application after 9 min in Ca^2+^-free bath solution). **b** Mean maximum responses ± SEM, expressed as percent of control response to ATP, of 28 SCs and 61 BCs (four VNO slices) in standard (*magenta and green columns*, respectively) and Ca^2+^-free bath solution (*light magenta* *and light green columns*, 3, 6, and 9 min in Ca^2+^-free bath solution, respectively). **c** ATP-induced [Ca^2+^]_i_ increases of SCs (*magenta*) and BCs (*green*; mean response ± SEM of all ATP-responsive cells of an acute slice of the VNO) were reversibly inhibited by depletion of intracellular Ca^2+^ stores with CPA (10 μM, *gray*-*shaded rectangle*; ATP application after 9 min in bath solution with CPA). **d** Mean maximum responses ± SEM, expressed as percent of control response to ATP, of 11 SCs (two slices) and of 51 BCs (four slices) in standard (*magenta and green columns*, respectively) and bath solution with CPA (*light magenta and light green columns*, 3, 6, and 9 min in bath solution with CPA, respectively). **e** ATP-induced [Ca^2+^]_i_ increases of SCs (*magenta*) and BCs (*green*; mean response ± SEM of all ATP-responsive cells of an acute slice of the VNO) were differentially affected by suramin (*SUR*, 200 μM, *gray*-*shaded rectangle*; ATP application after 9 min in bath solution with suramin). While ATP-induced [Ca^2+^]_i_ increases of SCs were unaffected by suramin, the responses of BCs showed a consistent reduction in bath solution with suramin. **f** Mean maximum responses ± SEM, expressed as percent of control response to ATP, of 10 SCs (two slices) and of 69 BCs (seven slices) in standard (*magenta and green columns*, respectively) and bath solution with suramin (*light magenta and light green columns*, 3, 6, and 9 min in bath solution with suramin, respectively). Statistical significance was tested using paired Student’s *t* test (*p< 0.05; **p< 0.01)

To find out whether metabotropic and/or ionotropic purinergic receptors are involved in the ATP-induced [Ca^2+^]_i_ increases in SCs and BCs, we first examined whether the presence of extracellular Ca^2+^ was essential for the responses to ATP (100 μM). In both cell populations, responses to ATP in Ca^2+^-free bath solution were the same or larger than those in standard solution (Fig. 4a). Similar results were obtained in 28 SCs and 61 BCs (four VNO slices, respectively) tested (Fig. 4b). On the other hand, depleting intracellular Ca^2+^ stores with CPA (10 μM) eliminated the intracellular Ca^2+^ responses to ATP almost completely in both cell populations (Fig. 4c). Virtually identical results were obtained in all of the 11 SCs and 51 BCs (two and four VNO slices, respectively) tested (Fig. 4d). Together, these data show that the ATP-induced [Ca^2+^]_i_ increases in SCs and BCs were mediated by Ca^2+^ release from CPA-sensitive intracellular stores, and that extracellular Ca^2+^ contributed little, if at all. Furthermore, the P2 receptor-antagonist suramin, applied at a concentration of 200 μM, differentially affected the ATP-induced [Ca^2+^]_i_ increases in SCs and BCs (Fig. 4e and f). Responses of SCs were not affected by suramin (10 SCs; two VNO slices). The mean ATP-induced [Ca^2+^]_i_ increase in BCs, in turn, was consistently reduced in the presence of suramin (69 BCs; seven VNO slices).

**Fig. 5:**
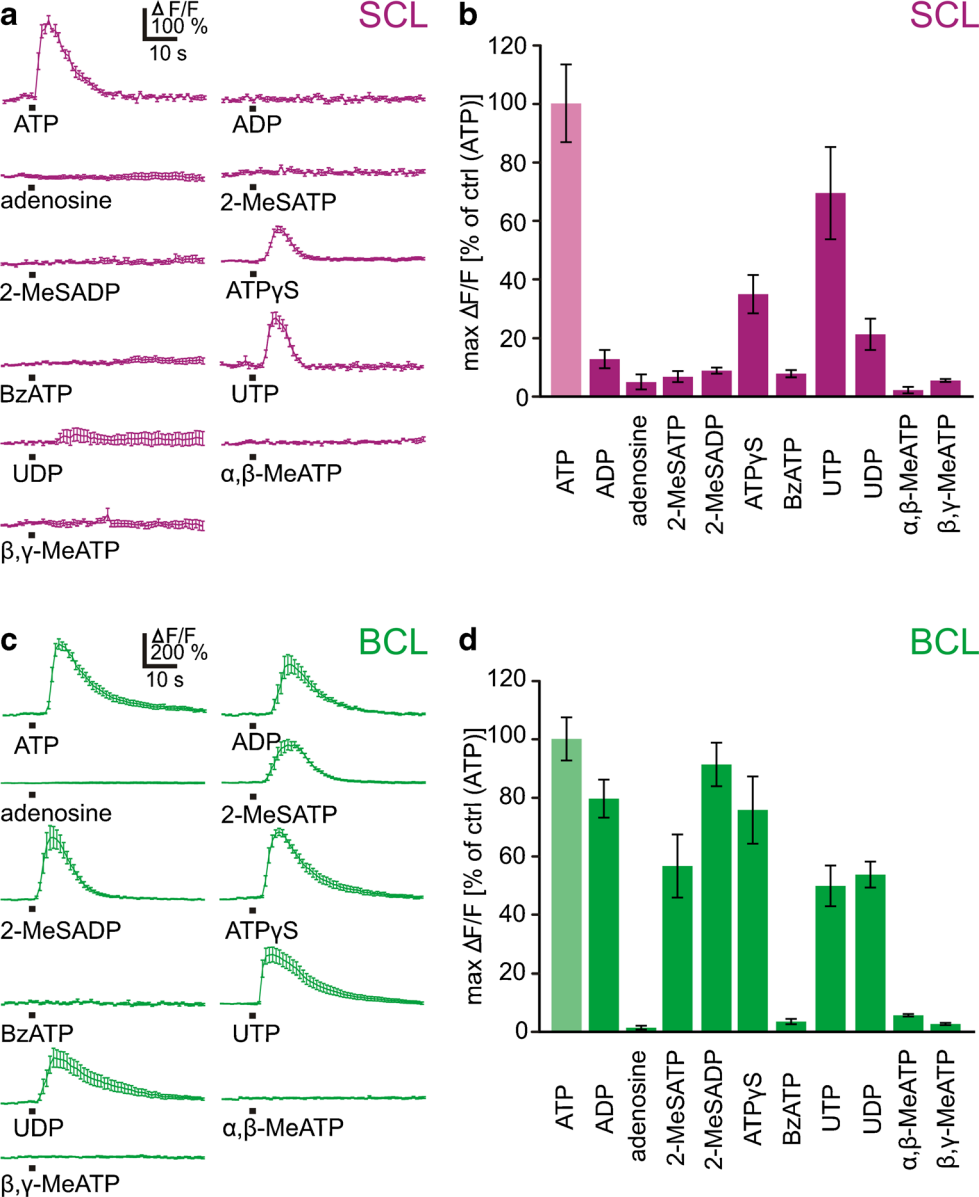
Differential responsiveness of supporting cells and basal cells to purinergic agonists. **a** [Ca^2+^]_i_ increases of SCs in response to application of various purinergic agonists (each 100 μM; mean response ± SEM of all ATP-responsive cells of VNO slices; traces originate from more than one slice). **b** Mean maximum responses ± SEM, expressed as percent of control response to ATP (*ATP* data from 131 SCs from 14 VNO slices; *ADP* 47/5; *adenosine* 29/3; *2*-*MeSATP* 55/6; *2*-*MeSADP* 64/7; *ATP*γ*S* 47/5; *BzATP* 50/6; *UTP* 80/8; *UDP* 47/5; α,β-*meATP* 9/2; β,γ-*meATP* 9/2). **c** [Ca^2+^]_i_ increases of BCs in response to application of various purinergic agonists (each 100 μM; mean response ± SEM of all ATP-responsive cells of VNO slices; traces originate from more than one slice). **d** Mean maximum responses ± SEM, expressed as percent of control response to ATP (*ATP* data from 429 BCs from 30 VNO slices; *ADP* 214/14; *adenosine* 122/7; *2*-*MeSATP* 163/11; *2*-*MeSADP* 291/19; *ATP*γ*S* 150/10; *BzATP* 105/7; *UTP* 189/13; *UDP* 188/13; α,β-*meATP* 81/4; and β,γ-*meATP* 81/4)

To obtain an indication on the specific purinergic receptor subtype(s) expressed by SCs and BCs, we tested the potency of a variety of purinergic agonists known to differentially activate purinergic receptors. [Ca^2+^]_i_ responses upon application of various purinergic agonists (each 100 μM) are shown in Fig. 5a and c, respectively. The bar graphs in Fig. 5 summarize the data obtained from 131 SCs (14 VNO slices; Fig. 5b) and 429 BCs (30 VNO slices; Fig. 5d). In addition to ATP, all SCs responded also to UTP and ATPγS, with an order of agonist potency of ATP > UTP > ATPγS. UTP was similarly potent as ATP (taken as 100 %), with mean maximum amplitudes of 69 ± 16 %. ATPγS was not as potent as ATP, with mean maximum amplitudes of 35 ± 7 %. All of the responsive SCs tested responded to these three purinergic agonists. In a subset of SCs, some irregular and faint responses were obtained also upon application of UDP and to a much lesser extent upon application of ADP. All other agonists tested, i.e., adenosine, 2-MeSADP, 2-MeSATP, BzATP, α,β-meATP, and β,γ-meATP were inactive. The response profile of BCs, on the other hand, was considerably broader. In addition to ATP, BCs responded to ADP, 2-MeSATP, 2-MeSADP, ATPγS, UTP, and UDP. 2-MeSADP, ADP, and ATPγS were nearly equipotent to ATP (taken as 100 %), with maximum amplitudes of 91 ± 7, 80 ± 7, and 76 ± 11 %, respectively. 2-MeSATP, UDP, and UTP were not as effective as ATP with maximum amplitudes of 57 ± 11, 54 ± 4, and 50 ± 7 %, respectively. The determined order of agonist potency was therefore: ATP ≥ 2-MeSADP ≥ ADP = ATPγS > UDP = UTP = 2-MeSATP. The nucleoside adenosine, BzATP, α,β-meATP, and β,γ-meATP were inactive. All of the individual BCs tested had the above mentioned response profiles to the applied agonists.

## Discussion

Purinergic signaling is known to be widespread throughout many tissues [22], including the central nervous system [23] and the special senses [12]. Among the special senses, the role of purinergic signaling in the olfactory system is certainly less studied than in the visual, auditory, or gustatory system (for a review see [12]). The olfactory system of tetrapod vertebrates is generally organized in different subsystems including the main and vomeronasal system, the septal organ and the Grueneberg ganglion [5]. The olfactory epithelia of all classes of tetrapods consist of the same three main cell types, i.e., receptor neurons, SCs, and BCs [5]. The presence of a purinergic system in the MOE has so far only been reported for amphibians [8, 9], and Murinae [10, 11, 18, 24]. The available information about purinergic receptors in the VNO is limited to two publications. Functional purinergic receptors have been shown to be expressed on isolated mouse VRNs [19], and an immunohistochemical study provided evidence for purinergic receptor expression in VRNs and SCs of the rat VNO [18]. In the present study, we describe the general cellular organization of the VNO of the amphibian *X. laevis* using tracing and immunohistochemical techniques and a BrdU incorporation assay, and characterize the vomeronasal purinergic system using functional calcium imaging.

### Cellular organization of the vomeronasal epithelium

As in the MOE [8, 9], antibodies against cytokeratin type II turned out to specifically stain SCs, but not VRNs and BCs of the VNO of larval *X. laevis*. Supporting cells form a columnar layer in the most apical part of the vomeronasal epithelium and send thin processes across the whole width of the VNO. The somata of VRNs, visualized by biocytin-backfills occupy an intermediate zone of the epithelium. Proliferating BCs, visualized by BrdU stainings, reside in the basalmost layer of the epithelium. Sparse BrdU-positive cells were also present in the most apical epithelial layer, showing that similarly, as in other vertebrates [25], also SCs of the *Xenopus* VNO are able to duplicate. The few BrdU-positive cells in the intermediate part of the epithelium, most probably represent newly formed immature VRNs. In the murine VNO, two types of BCs have been described. Marginally situated BCs have been shown to be responsible for the growth of the organ, BCs situated more centrally have been associated with neuronal replacement [26–28]. Whether different types of BCs exist also in the VNO of *Xenopus* will be tackled in a future, more specific, study. Together, these results show that the epithelium of the *Xenopus* VNO is generally structured as its MOE [8, 9].

### ATP-induced Ca^2+^ signaling in cells of the vomeronasal organ

Application of ATP to acute slices of the VNO induced strong [Ca^2+^]_i_ increases in both cells of the SCL and the BCL. As in SCs of the larval *Xenopus* and mouse MOE [8, 11], also the responses of SCs of the *Xenopus* VNO exhibited a wave-like propagation pattern. Cells of the VRNL showed virtually no response, strongly indicating that VRNs do not express purinergic receptors coupled to signaling pathways leading to increases in intracellular calcium concentration. Also ORNs of larval *Xenopus* MOE do not express purinergic receptors [7, 8]. The lack of purinergic receptors on VRNs could further be substantiated by calcium imaging in micro-Ruby backfilled VNO slices. Backfilled VRNs never responded to ATP, but responded to the application of bath solution with high K^+^. Increases of [Ca^2+^]_i_ upon stimulation with bath solution with high K^+^ indicates the presence of voltage-dependent Ca^2+^ channels, which is indicative of neurons [29]. This is in stark contrast to the murine olfactory system. Murine ORNs as well as VRNs have been shown to express purinergic receptors [10, 18]. The activation of purinergic receptors in mouse ORNs and VRNs has been shown to modulate their responsiveness to odorants [10, 19]. Of course, our study does not exclude that VRNs of the adult *Xenopus* olfactory system express purinergic receptors. The ATP-induced [Ca^2+^]_i_ increases in SCs and BCs persisted in the absence of extracellular Ca^2+^, but were virtually abolished after application of CPA, a specific inhibitor of the sarcoplasmic–endoplasmic reticulum Ca^2+^ ATPase [30], that leads to the depletion of intracellular Ca^2+^ stores. This, in turn, is a strong indication that the ATP-induced [Ca^2+^]_i_ increases in both cell types are almost exclusively due to the activation of metabotropic purinergic receptors coupled to store-dependent Ca^2+^ release, most probably P2Y receptor subtypes. P2Y receptors are a family of G protein coupled receptors present in a multitude of species [31]. Most studies, however, focused on mammalian P2Y receptors. The mammalian P2Y-receptor family comprises eight subtypes. Among these, P2Y_1_, P2Y_6_, and P2Y_12_ are activated mainly by nucleoside diphosphates, while P2Y_2_ and P2Y_4_ are selective for nucleoside triphosphates. P2Y_2_, P2Y_4_, and P2Y_6_ subtypes are activated by both purines and pyrimidines. P2Y_1_, P2Y_11_, and P2Y_12_ subtypes, in turn, show selectivity for purines. More specifically, 2-MeSADP is the most potent agonist at P2Y_1_, P2Y_12_, and P2Y_13_ receptors, ATP and UTP are almost equipotent at P2Y_2_ and P2Y_4_ receptors, P2Y_6_ has the highest affinity for UDP; P2Y_11_ is best activated by ATPγS and UDP-glucose selectively activates P2Y_14_ (for reviews see [22, 32, 33]).

### Identification of purinergic receptor subtypes expressed by supporting and basal cells

In an attempt to determine the P2Y receptor subtype(s) involved, we compared the effect of ATP with those of a number of putative purinergic agonists. In addition to ATP, only UTP and ATPγS activated all SCs of the VNO. Among all P2Y receptors, only P2Y_2_ and P2Y_4_ subtypes have been reported to be preferentially and almost equipotently activated by ATP and UTP [22, 32]. Furthermore, ATPγS is known to activate P2Y_2_ and P2Y_4_ receptors with a lower potency than ATP and UTP [34, 35]. These data are a good indication that SCs of the *Xenopus* VNO express P2Y_2_/P2Y_4_-like receptors. Some SCs additionally responded to UDP and to a much lesser extent to ADP. These responses, however, were quite irregular and therefore not comparable to the responses to ATP, UTP, and ATPγS. It is known that immature and mature cells often express different purinergic receptor subtypes [36]. Therefore, the observed responses to UDP and ADP could come from immature SCs [25], transiently expressing a different set of purinergic receptors if compared to mature SC. All responsive BCs of the VNO were activated by ATP, ADP, 2-MeSATP, 2-MeSADP, ATPγS, UTP, and UDP. The agonist profile and the order of agonist potency (ATP ≥ 2-MeSADP ≥ ADP = ATPγS > UDP = UTP = 2-MeSATP) of the P2Y receptor(s) of BCs does not match with any of the known agonist profiles of individual mammalian P2Y receptors [22, 32, 33], and is also not consistent with the agonist profile of P2Y_8_, a purinergic receptor cloned from *Xenopus* [37]. The fact that BCs responded to nucleoside diphosphates and triphosphates, and their inability to discriminate between purines and pyrimidines is a good indication that they express multiple P2Y subtypes. Of course, we cannot exclude that SCs and BCs express one or more not yet identified *Xenopus*-specific P2Y subtype(s). The observation that the P2X receptor agonists α,β-meATP and β,γ-meATP, as well as the P1 receptor agonist adenosine [22, 38] were ineffective on both cell types (SCs and BCs) further excludes an involvement of P2X and P1 receptors in purinergic signaling of the VNO. The non-selective P2 receptor-antagonist suramin [32, 38], slightly attenuated ATP-induced [Ca^2+^]_i_ increases solely in BCs. The responses of SCs were not affected at all. It has been shown that high concentrations of suramin affect all P2Y subtypes except P2Y_4_ [32]. Together with the agonist results, this indicates that P2Y_4_ is very likely to be among the P2Y subtypes expressed by BCs, and that SCs might express functional P2Y_4_ rather than P2Y_2_ subtypes. Other possible purinergic receptor subtypes expressed by BCs could be P2Y_1_, P2Y_6_, P2Y_12_, and P2Y_13_ which are predominantly activated either by nucleoside diphosphates or 2-MeSADP [22, 32, 33].

Taken together, the present study is the first systematic investigation of the purinergic system of an amphibian VNO. We provide evidence that functional P2Y receptors are expressed by non-neuronal cells (SCs and BCs), but not by receptor neurons. These data show that SCs and BCs of the *Xenopus* VNO and MOE express the same or very similar purinergic receptor subtypes (cf. [8, 9]), suggesting that purinergic signaling in both olfactory organs regulates similar physiological processes. In the *Xenopus*, MOE purinergic signaling has been shown to regulate cell proliferation of olfactory progenitor cells [9]. It remains to be shown if the purinergic system of the VNO serves a similar purpose. The similarity with the responses in mouse SCs [11], in turn, allows the speculation that some nucleotide-induced effects appear to be a common feature of olfactory organs of diverse vertebrate species. Unlike in the murine MOE and VNO [10, 18, 19], receptor neurons of both olfactory organs of *Xenopus* appear not to express purinergic receptors. This, in turn, clearly shows that also some fundamental differences exist between the purinergic systems of the olfactory organs of different vertebrate species.
